# Neurobrucellosis Presenting with Unilateral Abducens Nerve Palsy

**Published:** 2017

**Authors:** Mohsen ANDISHEH, Susan AMIRSALARI, Mohammad TORKAMAN, Marzieh SABZECHIAN, Shahla AFSHARPAIMAN

**Affiliations:** 1 New Hearing Technologies Research Center, Baqiyatallah University of Medical Sciences, Tehran, Iran.; 2 Pediatric Neurology Department, New Hearing Technologies Research Center, Baqiyatallah University of Medical Sciences, Tehran, Iran.; 3 Pediatrics Department, Baqiyatallah University of Medical Sciences, Tehran, Iran.; 4 Pediatrics Department, Health Research Center, Baqiyatallah University of Medical Sciences, Tehran, Iran.

**Keywords:** Neurobrucellosis, Abducens nerve palsy, Neuroimaging

## Abstract

One of the rare complications of brucellosis is neurobrucellosis. There have been numerous reports showing clinical forms of brucellosis affecting CNS, such as cranial nerve involvement, myelitis, vascular disease, radiculoneuritis, meningitis, meningoencephalitis, and demyelinating disease. In this case report, we introduce a 2.5 yr old girl with unilateral abducens nerve palsy referred to Baghiyatallah Hospital Outpatient Clinic, Tehran, Iran in June 2015.

## Introduction

Brucella, an intercellular bacterium causing chronic granulomatous infection, resembles tuberculosis and demands a compounded and extensive antibiotic treatment (1). Brucellosis is a zoonotic disease, endemic to certain geographic locations of the world including the Middle East, Central Asia, and Mediterranean countries (2, 3). In humans, this infection is acquired from animals. It occurs because of consuming infected dairy products, coming to direct contact with infected animal organs, and inhaling aerosolized infected particles (1, 4) .

Neurobruscellosis (NB) is reported to affect 5%-10% of brucellosis cases with presenting symptoms of the peripheral and central nervous system (5). Several clinical symptoms and imaging anomalies of NB can imitate other neurologic diseases (2, 6).

## Case report

The patient was a 2.5-yr-old girl referred to Baghiyatallah Hospital Outpatient Clinic, Tehran, Iran in June 2015 with left eye isotropia for 3 days, fever and occasional vomiting for 20 days. She was treated by amoxicilline-clavulanic acid, acetaminophen and ibuprofen. She had the history of consuming fresh un-posturized milk.

**Fig 1:**
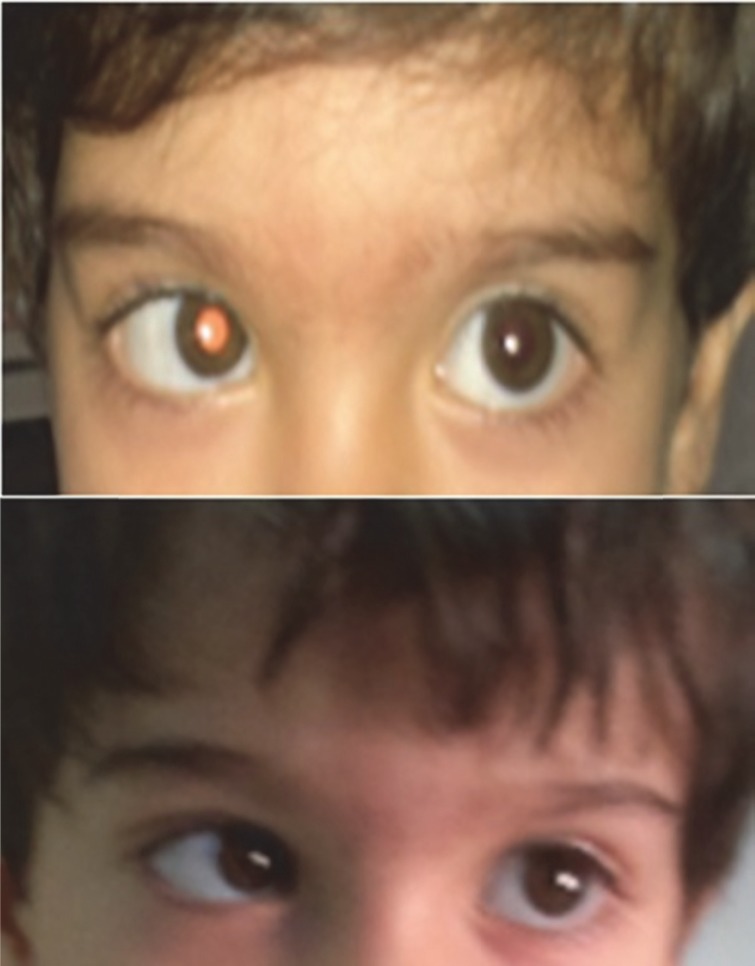
Left side abducens nerve palsy

In physical exam, we found low grade fever (38 degree of centigrade) and internal deviation of left eye (Figure 1). Other systemic and neurologic exams were normal. Informed consent was taken from patients parents and Ethics Committee of the hospital approved the study.

**Neuroimaging results**

**Fig 2:**
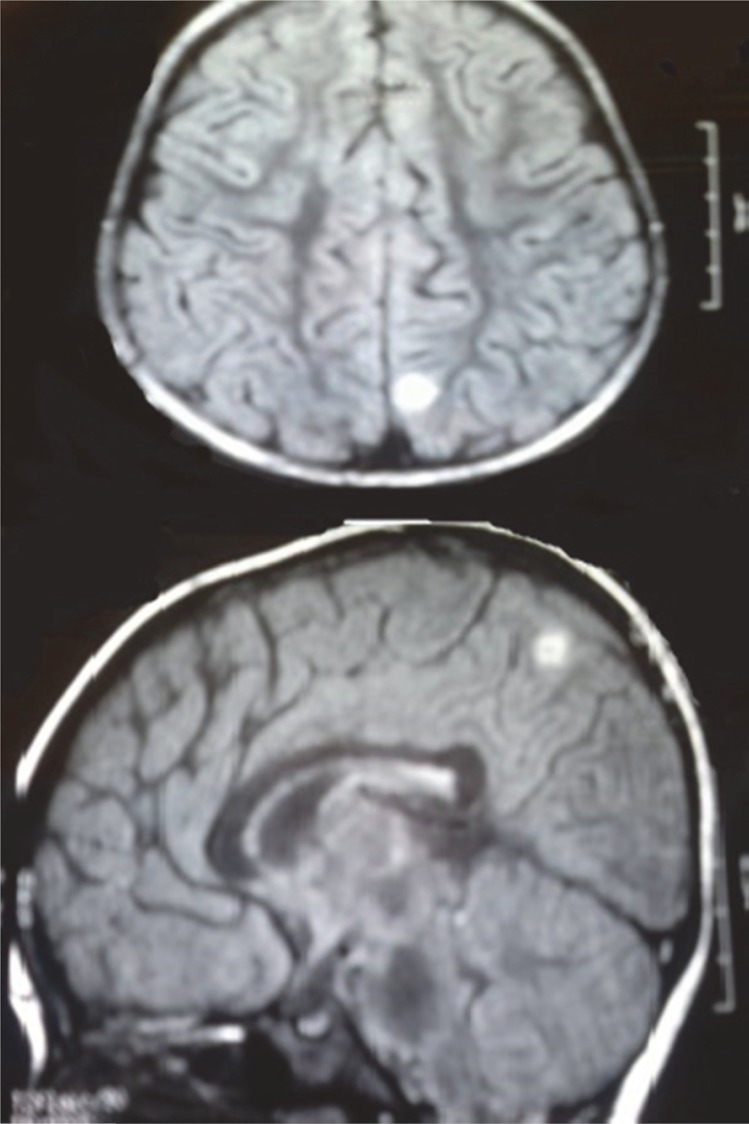
Round hyperintense lesion at left parasagitalparieto-occipital area in brain MRI

**Fig 3:**
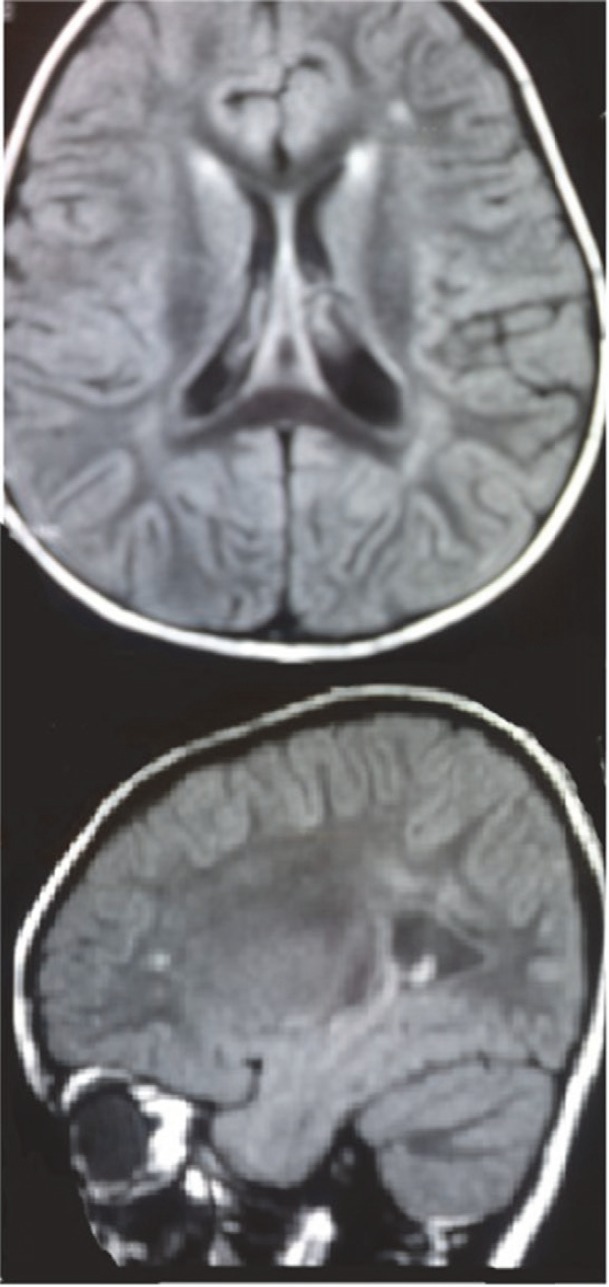
Round hyperintense frontal subcortical lesion in brain MRI

Brain MRI: There was a round hyperintense lesion at parasagital cortex of left parieto-occipital area on T1-weighted images (Figure 2). There was another small hyper intense lesion at frontal subcortical white matter, near the anterior horn of left lateral ventricle on T1-weighted images as well (Figure 3). Orbital MRI was normal.

**Laboratory findings**

CBC: WBC= 11600 (Poly: 44.2%, Lymph: 46%, Mono: 9%), Hb= 10.4, HCT=31.6, Platelet= 244000

ESR=17

CRP= Negative

Widal test: Negative

Wright Test: positive(titer 1.640)

2ME: Positive (titer 1.640)

Coombs wright: Positive (titer1.1280)

Toxoplasma- IgM: Borderline (1.1)

Toxoplasma- IgG: Negative

CMV (IgM): Negative

EBV(IgM): Negative

HIV (Ag and Ab): Negative

Hbs-Ab: Positive (vaccination has been performed)

CSF Analysis: RBC=5, WBC= 8[Polymorph: 20%,

Lymph: 80%], Glucose: 47 mg/dL,

Protein: 43.6 mg/dL

CSF Culture: Negative

Blood Culture: Negative

Urine Culutre: Negative

ANA= Negative

C3, C4, CH50= Normal

Treatment was started by the diagnosis of neurobrucellosis with the following protocol:

1) Intravenous gentamycine (30 mg BID) for 10 days, 2) Oral rifampin (150 mg per day) for 6 weeks, 3) Oral trimethoprim-sulfimetoxazole (200-40 mg TDS) for 6 weeks.

Fever was discontinued 3 days later and she was discharged from hospital 10 days after starting the antibiotic protocol.

In outpatient follow up visiting 7 days, six and nine months later, there was no fever recurrence or eye deviation. Physical and neurologic exams were normal.

Lab findings after six- months were wright test: 1.80, 2ME: 1.40 and after nine- months were wright test: Negative, 2ME: negative.

## Discussion

Brucella is considered as a crucial human pathogen, and is local to the under developed countries of the world.

Hematogenous dissemination may happen because of ingesting contaminated products, resulting in the absorption of the bacteria by the reticuloendothelial system, and ultimately, involvement of other organs (7).

Brucellosis is a disease that manifests itself in various forms, although fever has been a consistent feature, strongly unpleasant odor and constitutional symptoms generally appear as a result. Mostly, hepatosplenomegaly and lymphadenopathy can be present. Hepatitis, osteoarticular disease and genitourinary system involvement are commonly seen in brucellosis but central nervous system involvement is associated only in 5%-7% of cases most studied (1).

Neurobrucellosis is rarely seen in children, the symptoms may include fever, headache, vomiting, fatigue, depression, back pain, muscle tension and spasms. In addition to such symptoms, meningeal signs, absent deep tendon reflexes (DTR) or upward plantar reflex plus increased DTR and other symptoms of systemic brucellosis can be seen. Factors that could deteriorate the condition include sensorial or motor anomalies at different degrees, cranial nerve retention, convulsions, cerebellar dysfunction, coma and brain abscesses (8, 9). Development of NB is likely at any stage of the disease (5). Reports have shown numerous clinical forms of brucellosis affecting the CNS.

Such forms may include radiculoneuritis, myelitis, cranial nerve involvement, meningoencephalitis, and demyelinating or vascular disease (3, 5, 7, 10).

Chronic meningoencephalitis is among the most common forms of NB that affects the CNS, in which high protein levels and increased lymphocytes are detected in the CSF (11). Due to the slow-growing property of Brucella bacterium, CSF and blood cultures can show negative results. Therefore, serological methods are generally carried out to optimize the diagnosis. Detecting Brucella antibody in CSF is conclusive (4). The major clinical appearance of NB in children is depicted as acute meningitis or meningoencephalitis. Usually, in CSF assessment, brucellosis may not be included thus, it could make the diagnosis of the condition difficult (12, 13). Radiologic correlation in NB has been claimed variable (2). 

The imaging results of NB are categorized into four groups: normal, inflammation, abnormal white matter intensities, and vascular abnormalities. Demyelination of CNS is considered as a rare manifestation of NB (5, 11, 14).

The white matter alterations in NB were a consequence of demyelination; also confirmed by pathologic study (15). This claim was bolstered with autopsy evidence that demyelination in a patient with NB resembled the lesions of multiple sclerosis (6, 16). The underlying reason for the changes in white matter is yet to be known, but they may be resulted from an autoimmune reaction (2). Not only the white matter involvement of NB resembles that of MS or Vasculitis, but also it can imitate that of other inflammatory or infectious diseases such as acute disseminated encephalomyelitis or Lyme disease (2, 17). Due to basal meningitis, involvement of one or more cranial nerves is observed in more than half of NB cases. The vestibulocochlear nerve is the cranial nerve most commonly involved in NB (3). It is very rare to see isolated cranial nerve involvement in NB; only a few isolated abducens nerve palsies (6, 18, 19) have been reported. The pathogenesis of abducens nerve palsy is hypothetical. Possible etiologies include extension of meningeal infection and possible vasculitis processes (18).

**In conclusion, **NB, as a disease, can be treated with a favorable result. The diagnosis of this disease is contingent mainly upon high clinical attention in endemic countries. It is presented diversely in clinical or radiological diagnoses, particularly in young patients with neurological abnormalities. Imaging results of NB can potentially be misleading as they are varied and can imitate properties of other demyelinating, infectious, or inflammatory conditions.

## References

[1] Pappas G, Akritidis N, Bosilkovski M, Tsianos E (2005). Brucellosis. N Engl J Med.

[2] Al-Sous MW, Bohlega S, Al-Kawi MZ, Alwatban J, McLean DR (2004). Neurobrucellosis: clinical and neuroimaging correlation. AJNR Am J Neuroradiol.

[3] Bingöl A, TogayIsikay C (2006). Neurobrucellosis as an exceptional cause of transient ischemic attacks. Eur J Neurol.

[4] Adaletli I, Albayram S, Gurses B (2006). Vasculopathic changes in the cerebral arterial system with neurobrucellosis. Am J Neuroradiol.

[5] Shakir RA, Al-Din AS, Araj GF, Lulu AR, Mousa AR, Saadah MA (1987). Clinical categories of neurobrucellosis A report on 19 cases. Brain.

[6] Esra Özkavukcu, Zeynep Tuncay, Ferda Selçuk, İlhan Erden (2009). .An unusual case of neurobrucellosis presenting with unilateral abducens nerve palsy: clinical and MRI findings.

[7] Tena D, Gonzáles-Praetorius A, LópezAlonso A, Peña JL, Pérez-Pomata MT, Bisquert J (2006). Acute meningitis due to Brucella spp. Eur J Pediatr.

[8] Rangel Guerra R, Martinez HR, Leon Flores L (Rev Inwest Clin. 1982). Neurobrucellosis. Report of five cases and literature review.

[9] Mugerwa RD, D’Arbela PG (1976). Brucella meningitis; a case report and review of the literature. East African Med J.

[10] Bashir R, Al-Kawi MZ, Harder EJ, Jinkins J (1985). Nervous system brucellosis: diagnosis and treatment. Neurology.

[11] Al Deeb SM, Yaqub BA, Sharif HS, Phadge JG (1989). Neurobrucellosis: clinical characteristics, diagnosis, and outcome. Neurology.

[12] Lubani MM, Dudin KI, Araj GF, Manandhar DS, Rashid FY (1989). Neurobrucellosis in children. Pediatr Infect Dis J.

[13] Santini C, Baiocchi P, Berardelli A, Venditti M, Serra P (1994). A case of brain abscess due to Brucella melitensis. Clin Infect Dis.

[14] Koussa S, Chemaly R (2003). Neurobrucellosis presenting with diffuse cerebral white matter lesions. Eur Neurol.

[15] Fincham RW, Sahs AL, Joynt RJ (1963). Protean manifestation of nervous system brucellosis Case histories and a wide variety of clinical forms. JAMA.

[16] Marconi G (1966). Su un caso di sclerosis multipla acuta insorta dopo un’infezione da Brucella abortus. Riv Patol Nerv Ment.

[17] Bussone G, La Mantia L, Grazzi L, Lamperti E, Salmaggi A, Strada L (1989). Neurobrucellosis mimicking multiple sclerosis: a case report. Eur Neurol.

[18] KarakurumGöksel B, Yerdelen D, Karataş M (2006). Abducens nerve palsy and optic neuritis as initial manifestation in brucellosis. Scand J Infect Dis.

[19] Yilmaz M, Ozaras R, Mert A, Ozturk R, Tabak F (2003). Abducent nerve palsy during treatment of brucellosis. Clin Neurol Neurosurg.

